# Asiatic acid attenuates renin-angiotensin system activation and improves vascular function in high-carbohydrate, high-fat diet fed rats

**DOI:** 10.1186/s12906-016-1100-6

**Published:** 2016-04-27

**Authors:** Putcharawipa Maneesai, Sarawoot Bunbupha, Upa Kukongviriyapan, Parichat Prachaney, Panot Tangsucharit, Veerapol Kukongviriyapan, Poungrat Pakdeechote

**Affiliations:** Department of Physiology, Faculty of Medicine, Khon Kaen University, Khon Kaen, 40002 Thailand; Department of Anatomy, Faculty of Medicine, Khon Kaen University, Khon Kaen, 40002 Thailand; Department of Pharmacology, Faculty of Medicine, Khon Kaen University, Khon Kaen, 40002 Thailand

**Keywords:** Asiatic acid, Metabolic syndrome, Vascular function, Rennin-angiotensin system

## Abstract

**Background:**

In the rat model of high carbohydrate, high fat (HCHF) diet-induced metabolic syndrome (MS), previous studies have found that asiatic acid has an antihypertensive effect. In this study, we investigated effects of asiatic acid on vascular structure, vascular function and renin-angiotensin system (RAS) in HCHF diet-induced MS rats.

**Methods:**

Male Sprague–Dawley rats were divided into three treatment groups for the 15 week study: a control group fed a normal diet, a MS group fed HCHF diet plus 15 % fructose in their drinking water for 15 weeks, and an asiatic acid treated group that received a HCHF diet plus fructose for 15 weeks and also received orally administered asiatic acid (20 mg/kg BW/day) for the final 3 weeks. Vascular structure and function were investigated. AT_1_ receptor expression in aortic tissues and eNOS protein expression in the mesenteric arteries were detected. The levels of serum angiotensin (Ang) II, angiotensin converting enzyme (ACE) and plasma norepinephrine (NE) were measured. The differences among treatment groups were analyzed by one-way analysis of variance (ANOVA) followed by post-hoc Bonferroni tests.

**Results:**

At the end of the study, all rats fed a HCHF diet exhibited signs of MS including, hypertension, dyslipidemia and insulin resistance. Vascular remodeling in large and small arteries, overexpression of AT_1_ receptor, and high levels of serum Ang II and ACE were also observed in MS group (*p* < 0.05). Contractile responses to sympathetic nerve stimulation were enhanced relating to high plasma NE level in MS rats (*p* < 0.05). The response to exogenous NE was not changed in the mesenteric bed. Vasorelaxation responses to acetylcholine were blunted in thoracic aorta and mesenteric beds, which is consistent with downregulation of eNOS expression in MS rats (*p* < 0.05). Restoration of metabolic alterations, hemodynamic changes, RAS and sympathetic overactivity, increased plasma NE, endothelium dysfunction, and downregulation of eNOS expression was observed in the asiatic acid treated group (*p* < 0.05). However, asiatic acid failed to alleviate vascular remodeling in MS rats.

**Conclusion:**

Our findings suggest that the observed antihypertensive effect of asiatic acid in MS rats might be related to its ability to alleviate RAS overactivity and improve vascular function with restoration of sympathetic overactivity.

**Electronic supplementary material:**

The online version of this article (doi:10.1186/s12906-016-1100-6) contains supplementary material, which is available to authorized users.

## Background

Metabolic syndrome (MS) is a group of metabolic abnormalities including abdominal obesity, dyslipidaemia, hyperglycaemia, and hypertension that are risk factors for cardiovascular disease (CVD) and diabetes mellitus type 2 (DM2) in human [1]. There are many combinations of diets, including high fructose, high carbohydrate and high fat diets, used in rodents to mimic MS [2, 3]. High carbohydrate-high fat (HCHF) diets have been shown to induce metabolic syndrome (MS) in rats. MS affected rats regularly show insulin resistance, impairment of glucose tolerance, dyslipidemia and high blood pressure [4, 5]. The development of hypertension in diet-induced MS in rats has been reported, along with structural and functional vascular alterations [4, 6]. Thickening of the media layer of mesenteric arteries, indicating vascular remodeling, has been seen in fructose-induced metabolic syndrome [7]. Panchal and coworkers (2011) reported that HCHF diet-induced MS rats exhibited hypertension and a reduction of the vasorelaxation response to acetylcholine [8]. Microvascular dysfunction has also been reported to be an important factor that contribute to the development of hypertension in MS rats [9]. Several mechanisms have been proposed in the pathogenesis of endothelial dysfunction, such as decrease in nitric oxide (NO) bioavailability and increases in oxidative stress and inflammation [10, 11].

Sympathetic nerve overactivity strongly associated with hyperinsulinemia has been found in metabolic syndrome human patients [12] and diet-induced MS rats [13, 14]. Numerous studies suggest that in mesenteric vascular beds isolated from fructose-induced insulin resistance rats, hyperinsulinemia causes hypertension by enhancing contractile responses to sympathetic nerve activity [15]. In pithed rats without a central modulation, chronic hyperinsulinemia can be induced by fructose-drinking facilitated pressor response to sympathetic nerve stimulation, a response that might contribute to the development of hypertension [16].

Furthermore, it is well known that the renin-angiotensin system is important for blood pressure regulation. Angiotensin (Ang) II induces high blood pressure through its receptor, the type 1 Ang II receptor (AT_1_ receptor), which mediates vasoconstriction [17]. It has been reported that renin-angiotensin system is activated in MS [18]. The association between high activity of the renin-angiotensin system and impairment of insulin sensitivity is evident in MS [19]. Several previous studies showed that the renin-angiotensin system plays a crucial role in the development of hypertension in high fructose fed rats, since an increase in plasma Ang II [20–22] and AT_1_ receptor mRNA levels were reported in fructose-induced hypertensive rats [23]. Thus, we hypothesize that the improvement of insulin resistance, sympathetic nerve and renin-angiotensin system overactivity along with vascular remodeling and function could alleviate high blood pressure and alleviate cardiovascular complications in diet-induced MS rats [5, 24, 25].

Asiatic acid is a triterpenoid compound derived from *Centella asiatica*. Numerous studies demonstrate the biological effects of asiatic acid. Asiatic acid has been reported to have antioxidative and anti-inflammatory activities in hydrogen peroxide (H_2_O_2_) induced injury performed in human bronchial epithelial cells [26]. Antihyperlipidemic and antidiabetic activities of asiatic acid in streptozotocin-induced diabetic rats have also been proposed [27]. Another recent study showed that asiatic acid reduced blood pressure, improved vascular function via restoring endothelial nitric oxide synthase (eNOS) and p^47phox^ expression in L-NAME hypertensive rats [28]. In rat models of HCHF diet-induced MS, previous studies found antihypertensive, anti-inflammatory and antioxidant effects of asiatic acid [5]. However, there is no information about the effects of asiatic acid on vascular structure and function in HCHF induced MS rats. In the present study, we investigated whether asiatic acid supplementation could alleviate vascular alterations and the renin-angiotensin system in HCHF diet-induced MS rats.

## Methods

### Drugs

Asiatic acid, ethylenediaminetetraacetic acid (EDTA), norepinephrine, acetylcholine, phenylephrine, sodium nitroprusside and capsaicin were obtained from Sigma-Aldrich (St. Louis, MO, USA) (purity *>*95 %). All other chemicals used were analytical grade quality.

### Animals

Male Sprague–Dawley rats (220–240 g) were purchased from the National Laboratory Animal Center, Mahidol University, Salaya, Nakornpathom. Rats were maintained in an air-conditioned room (23 ± 2 °C) with a 12 h dark–light cycle at the Northeast Laboratory Animal Center. All procedures complied with the standards for the care and use of experimental animals and were approved by the Animal Ethics Committee of Khon Kaen University, Khon Kaen, Thailand (AEKKU 36/2555).

### Experimental designs

We conducted two separate sets of experiments in order to study vascular structure and vascular function. After one week of acclimatization, the animals were randomly divided into 3 study groups: group 1, the normal control group (C; *n* = 14) ate a standard chow diet (ChareonPokapan Co. Ltd., Thailand) and drinking tap water, group 2 (MS; *n* = 14) ate an HCHF diet together with 15 % fructose in drinking water for 15 weeks, and group 3 (MS + AA; *n* =14) received an HCHF diet with 15 % fructose in their drinking water for the 15 weeks and were treated with asiatic acid at dose 20 mg/kg BW per day for the final 3 weeks of the 15 week study period. The compositions of HCHF diet (Additional file 1) followed the method of Senaphan and coworkers [6] and the dose of asiatic acid was influenced by previous study [5, 28].

### Indirect measurement of blood pressure in conscious rats

Systolic blood pressure (SP) and heart rate (HR) of rats were measured weekly using non-invasive tail cuff plethysmography (IITC/Life Science Instrument model 229 and model 179 amplifier; Woodland Hills, CA, USA) to assess blood pressure changes throughout the 15 weeks of the study.

### Assessment of metabolic parameters

Rat body weight was assessed once a week. Blood samples were taken to analyze lipid profiles, which were measured by Clinical Chemistry Laboratory Unit of Faculty of Associated Medical Sciences, Khon Kaen University, Thailand. Fasting blood glucose (FBG) was measured using a glucometer (Roche Diagnostics Australia Pty. Ltd., Sydney, Australia). Insulin level was measured using a Rat Insulin ELISA Kit (Millipore Corporation, Billerica, MA, USA). Homeostasis Model Assessment of Insulin Resistance (HOMAR-IR) score was used as an index of insulin resistance [29] and was calculated using Equation 1:

1 $$ \mathrm{HOMA}\hbox{-} \mathrm{I}\mathrm{R} = \left(\mathrm{fasting}\ \mathrm{glucose}\ \left(\mathrm{mmol}/\mathrm{L}\right) \times \mathrm{fasting}\ \mathrm{insulin}\ \left(\upmu \mathrm{I}\mathrm{U}/\mathrm{mL}\right)\right)/22.5 $$

### Hemodynamic assessment

Hemodynamic parameters were determined at the end of study. Briefly, rats were anesthetized by intra-peritoneal administration of pentobarbital-sodium (60 mg/kg) and placed on a heating pad. A polyethylene tube was inserted in to the femoral artery for direct blood pressure measurement. SP, diastolic blood pressure (DP), mean arterial pressure (MAP) and HR were continuously monitored by a way of pressure transducers and recorded using the Acknowledge Data Acquisition and Analysis Software (BIOPAC Systems Inc., California, USA). Subsequently, the abdominal aorta was carefully separated from the abdominal vein, cleaned of connective tissue and fitted with a flow probe to detect hindlimb blood flow (HBF, ml/min/100 g tissue) with an electromagnetic flowmeter (Carolina Medical Electronics, Inc., North Carolina, USA). Hindlimb vascular resistance (HVR, mmHg/ml/min/100 g tissue) was calculated by MAP divided by HBF. Thereafter, blood samples were collected from the abdominal aorta for biochemical assays.

### Morphometric measurement

The rats were euthanized, and mesenteric arteries (2^nd^ branches) and thoracic aorta (3^rd^–6^th^ vertebral level) were excised and cleaned of surrounding adipose and connective tissues. The samples were then perfused with 4 % paraformaldehyde and fixed in Bouin’s solution. They were then embedded in paraffin, and serial 5-μm-thick sections were cut and then stained with hematoxylin and eosin. Sections were captured with Digital sight DS-2MV light microscope (Nikon, Tokyo, Japan). Morphometric evaluations; wall thickness, cross-sectional area (CSA) and luminal diameters were performed on Image J software (National Institutes of Health, Bethesda, MD, USA).

### Biochemical measurements

Serum Ang II and angiotensin converting enzyme (ACE) concentrations were measured using Ang II EIA kit (St. Louis, MO, USA) and mouse ACE ELISA kit (St. Louis, MO, USA) respectively. Concentration of plasma norepinephrine (NE) was determined by HPLC with electrochemical detector (DECADE II, Waters, Milford, MA) using a commercials kit (RECIPE, Dessauerstraße 3, D-80992 Munich, Germany).

### Vascular function study

#### Experimental protocols in isolated mesenteric vascular beds

After hemodynamic assessment, the animals were sacrificed by exsanguination. Mesenteric vascular beds were carefully isolated and then placed on a stainless steel grid (7x5 cm) in a humid chamber. The preparations were perfused with physiological Kreb’s solution at a constant flow rate of 5 ml/min, using a peristaltic pump (07534–04, Cole-Palmer Instrument, Illinois, USA). Kreb’s solution is composed of the following (all numbers are in mM): NaCl 118, NaHCO_3_ 25, KCl 4.8, KH_2_PO_4_ 1.2, MgSO_4_.7H_2_O 1.2, CaCl_2_ 1.25 and glucose 11.1 [30]. The solution was maintained at 37 °C and continuously gassed with a 95 % O_2_ and 5 % CO_2_ gas mixture.

The mesenteric vascular beds were pretreated with a desensitizing agent, capsaicin (0.1 μM), for 20 min followed by 30 min washout period to facilitate a desensitization of vanilloid receptors and to cause a diminution of sensory neurotransmitters [31]. After the washout period, electrical field stimulation (EFS) (5–40 Hz, 90 V, 1 ms, for 30s at 5-min intervals) was performed. Contractile responses to EFS were detected as changes in mean perfusion pressure (mmHg) using a pressure transducer and data were recorded via the BIOPAC System (BIOPAC Systems Inc., California, USA). The preparations were allowed to equilibrate for 30 min before the next trial. After the resting period, the mesenteric vascular beds were injected with bolus doses of NE (0.15 nmol–15 nmol) to evaluate the contractile responses to exogenous NE. To determine vasoactive performance of resistance small arteries, methoxamine (5–7 μM) was added into Kreb’s solution to raise tone (70–90 mmHg above baseline). Subsequently, different doses of vasoactive agents, acetylcholine (ACh, 1 nM–0.01 μM) or sodium nitroprusside (SNP, 1 nM–0.01 μM) were injected through neoprene rubber tubing proximal to the tissue.

### Experimental protocols in isolated aortic rings

To assess vasoactive performance of the large arteries, the thoracic aorta was rapidly removed and cut into 2–3 mm long rings for tension measurement. Samples were mounted in 15 ml baths containing Krebs’ solution at 37 °C and gassed with 95 % O_2_ and 5 % CO_2_gas mixture. Isometric contractions were recorded with a resting tension of 1 g using a transducer connected to a 4-channel bridge amplifier and a PowerLab A/D converter and a PC running Chart v5 (PowerLab System, ADInstruments, Australia). ACh (0.01 μM–3 μM) induced endothelial mediated-relaxations and SNP (0.01 μM–3 μM) were assessed by pre-contracting with phenylephrine (10 μM) and relaxation expressed as percent of phenylephrine-induced contraction.

### Western blot analysis of eNOS and AT_1_ receptor protein expression

eNOS protein expression in the mesenteric artery and AT_1_ receptor protein expression in the thoracic aorta were measured using a Western blot following a previous described method [28] with some modifications. The vessels were homogenized and the proteins were electrophoresed on a sodium dodecylsulfate polyacrylamide gel electrophoresis system. Thereafter, the proteins were electrotransfered onto a polyvinylidenedifluoride membrane and blocked with 5 % skimmed milk in Tris-buffered saline (TBS) with 0.1 % Tween 20 for 2 h at room temperature followed by incubation overnight at 4 °C with mouse monoclonal antibodies to eNOS (BD Biosciences, CA, USA) or rabbit polyclonal antibodies to AT_1_ receptor (Santa Cruz Biotechnology, Inc., Santa Cruz, CA). After the incubation period, the membranes were washed with TBS and then incubated for 2 h at room temperature with horseradish peroxidase-conjugated secondary antibody. The blots were developed in AmershamTM ECLTM Prime solution (Amersham Biosciences Corp., Piscataway, NJ, USA), and densitometric analysis was performed using an ImageQuant™ 400 (GE Healthcare Life Sciences, Piscataway, NJ, USA). The intensity of eNOS or AT_1_ receptor bands was normalized to that of β-actin, and data were expressed as a percentage of the values determined in control group from the same gel.

### Statistical analysis

Data were expressed as mean ± S.E.M. Time-course effect of treatment on blood pressure was analyzed by two-way measured analysis of variance (ANOVA) with post-hoc Student Newman–Keul's test. The differences among treatment groups were analyzed by one-way analysis of variance (ANOVA) followed by post-hoc Bonferroni tests. A *p*-value of less than 0.05 was considered statistically significant.

## Results

### Effects of asiatic acid on blood pressure and heart rate in conscious rats

**Fig. 1:**
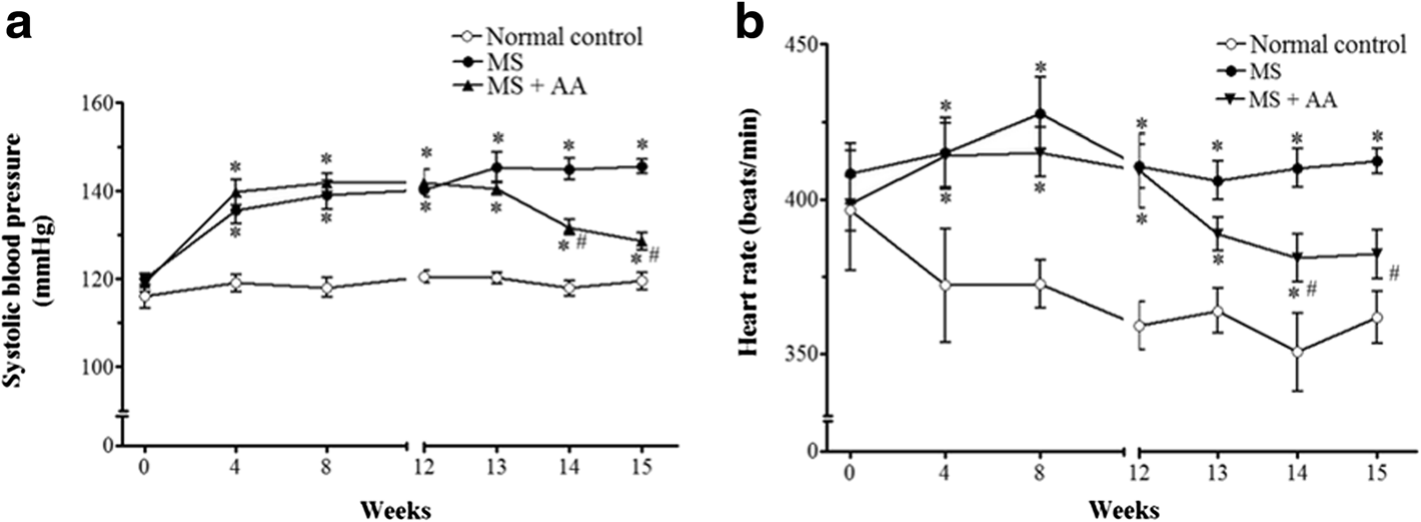
Effects of asiatic acid on blood pressure and heart rate in conscious rats. Systolic blood pressure (**a**) and heart rate (**b**) were measured monthly during the induction periods and weekly during the last 3 weeks of the treatment period. Values are mean ± SEM (*n* = 14 for each group), *p <* 0.05. * vs. control group, ^#^vs. MS group. Normal control = normal control rats, MS = metabolic syndrome rats, MS + AA = metabolic syndrome rats treated with asiatic acid

There were increases in SP (145.6 ± 1.7 vs. 119.5 ± 1.9 mmHg) and HR (412.2 ± 4 vs. 361.6 ± 8.3 beat/min) in MS rats comparing to that levels in control rats (*p* < 0.01). However, MS rats treated with asiatic acid for the last 3 weeks had a significant reduction of SP (128.5 ± 1.9 mmHg) and HR (382.2 ± 7.9 beat/min) when compared to those of untreated rats (*p* < 0.05; Fig. 1).

### Effects of asiatic acid on hemodynamic parameters

After 15 weeks of MS induction, SP, DP, MAP and HR in MS group were significantly increased when compared to those of the normal control group (*p* < 0.05). Treatment with asiatic acid lowered SP, DP, MAP and HR comparing to MS rats (*p* < 0.05; Table 1). Moreover, a marked reduction of HBF was found in MS group, which was associated with a significant increase in HVR compared to those of control group (*p* < 0.05; Table 1). Treatment with asiatic acid improved both HBF and HVR in MS + AA rats comparing to MS rats.

**Table 1:** Effects of asiatic acid on hemodynamic status and metabolic parameters

Parameters	Normal control	MS	MS + AA
SP (mmHg)	115.7 ± 2.1	149.2 ± 4.2*	130.2 ± 2.6*^,^**
DP (mmHg)	75.2 ± 1.9	100.9 ± 2*	88.5 ± 1.3*^,^**
MAP (mmHg)	89.2 ± 1.8	117.8 ± 2.5*	103.8 ± 1.7*^,^**
HR (beats/min)	351.3 ± 12.7	418.5 ± 11.4*	361.8 ± 10.1**
HBF (mL/min/100 g tissue)	7 ± 0.3	4.1 ± 0.1*	6.1 ± 0.3**
HVR (mmHg/mL/min/100 g tissue)	13.1 ± 0.5	29 ± 1.2*	17.6 ± 0.9*^,^**
Fasting blood glucose (mg/dL)	86.9 ± 2.5	117.6 ± 1.7*	90.6 ± 1.5**
Fasting serum insulin (ng/mL)	0.4 ± 0.1	4.7 ± 0.5*	1.9 ± 0.3*^,^**
HOMAR-IR index	1.9 ± 0.2	31.2 ± 3.3*	9.9 ± 1.5**
Cholesterol level (mg/dL)	52.2 ± 1.8	82.3 ± 2.3*	57.3 ± 2.4*^,^**
Triglyceride level (mg/dL)	28.6 ± 3.6	80.7 ± 5.8*	31.7 ± 1.7**
HDL-C level (mg/dL)	42.5 ± 1.6	18.2 ± 1.8*	31.2 ± 2.1*^,^**

*SP* systolic blood pressure, *DP* diastolic blood pressure, *MAP* mean arterial pressure, *HR* heart rate, *HBF* hindlimb blood flow, *HVR* hindlimb vascular resistance, *HOMA-IR*, homeostasis model assessment of insulin resistance, *HDL-C* high density lipoprotein cholesterol. Values are mean ± SEM (*n* = 14 for each group); *p < 0.05 compared to the control group; **p < 0.05 compared to the MS group

### Effects of asiatic acid on metabolic parameters

No significant difference in body weight was observed between experimental groups (490 ± 7, 475.2 ± 9.2 and 462.3 ± 10.5 g in control, MS and MS + AA groups, respectively). Rats fed the HCHF diet showed significant increases in fasting blood glucose, insulin levels and HOMAR-IR index comparing to those of the control group (*p* < 0.05; Table 1). Moreover, MS rats exhibited abnormal lipid profiles including increases total cholesterol and triglycerides levels and decreased HDL-C level in serum compared to those of control rats (*p* < 0.05; Table 1). Interestingly, treatment with asiatic acid 20 mg/kg BW for 3 weeks significantly reduced fasting blood glucose, insulin levels and HOMAR-IR index and also improved total cholesterol, triglycerides and HDL-C levels comparing to those of MS rats (*p* < 0.05; Table 1).

### Effects of asiatic acid on vascular remodeling and AT_1_ receptor protein expression

**Fig. 2:**
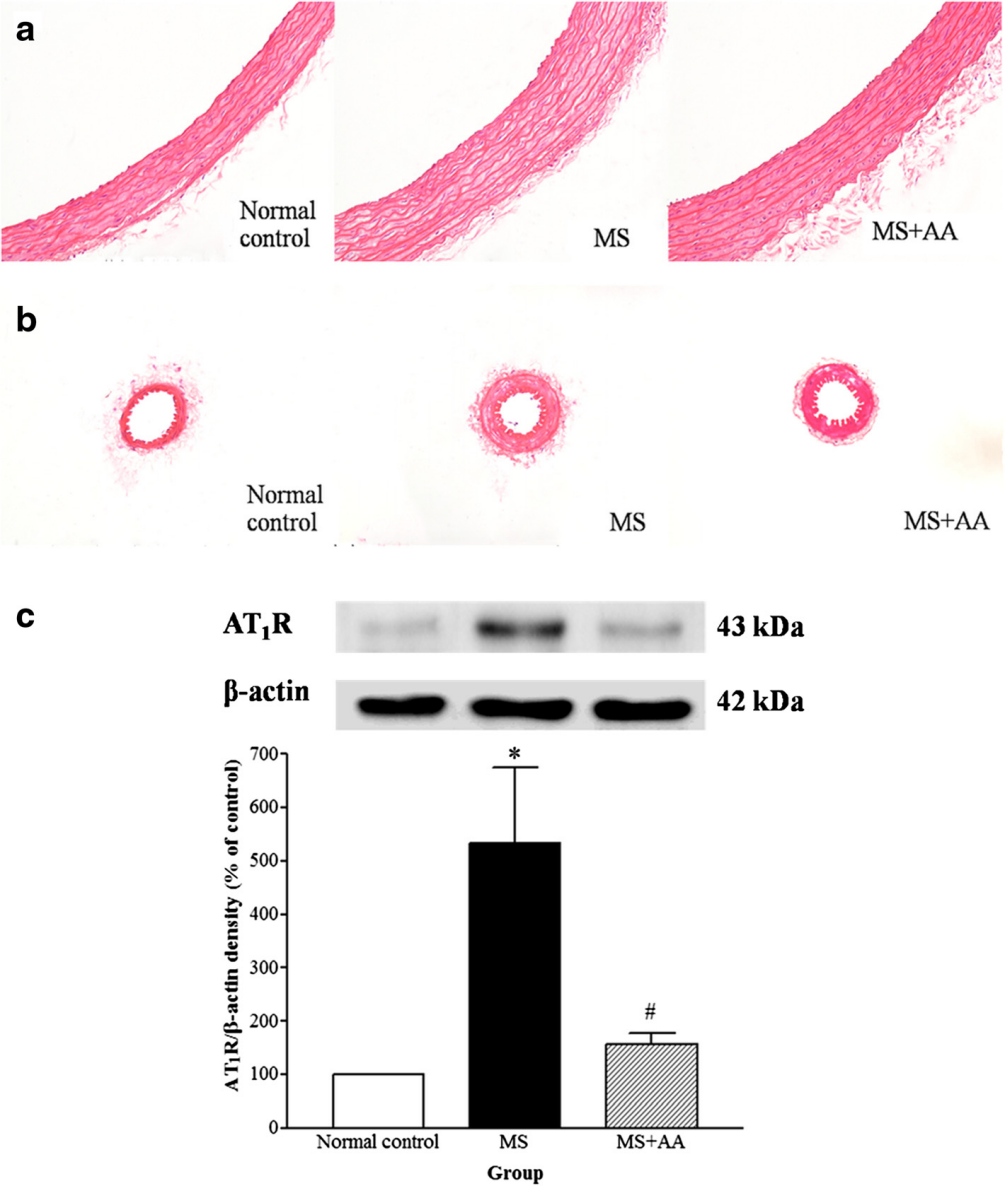
Effects of asiatic acid on vascular remodeling and AT_1_ receptor protein expression in thoracic aorta. *Top panel*, representative photographs of thoracic aorta (**a**) and 2^nd^ order of mesenteric artery samples (**b**) stained by H&E (original magnification = X 20) (*n* = 7) and AT_1_ receptor protein expression in thoracic aorta (*n* = 4) (**c**) of all experimental groups. Values are mean ± SEM, *p <* 0.05. * vs. control group, ^#^vs. MS group. Normal control = normal control rats, MS = metabolic syndrome rats, MS + AA = metabolic syndrome rats treated with asiatic acid

Vascular wall hypertrophy was observed in the aorta (Fig. 2a) and the mesenteric artery of MS rats (Fig. 2b), as evidenced by a significant increase in vascular wall thickness, CSA and lumen diameters compared to those of control rats (*p* < 0.01; Table 2). Asiatic acid did not reverse the vascular abnormalities in either the aorta or the mesenteric artery. Furthermore, there was up-regulation of AT_1_ receptor protein expression in the thoracic aorta compared to that of the control group (*p* < 0.001). This overexpression of AT_1_ receptor reverted to normal in MS rats treated with asiatic acid (*p* < 0.01; Fig. 2c).

**Table 2:** Effects of asiatic acid on morphologic changes in the thoracic aorta and 2^nd^ order of mesenteric artery

Parameters	Normal control	MS	MS + AA
Wall thickness_a_ (μm)	113.7 ± 4.4	133.9 ± 5.3*	131.1 ± 4.5*
CSA_a_ (x10^3^μm^2^)	658.6 ± 26.4	802 ± 58.8*	787.8 ± 30.4*
Luminal diameter_a_ (μm)	1723.7 ± 44.1	1527.3 ± 41.2*	1589.7 ± 20.7*
Wall thickness_m_ (μm)	27.8 ± 1.6	33.2 ± 1*	29.4 ± 1.2*
CSA_m_ (x10^3^μm^2^)	10.5 ± 0.8	13.5 ± 0.7*	13 ± 0.4*
Luminal diameter_m_ (μm)	136.3 ± 3.1	84.1 ± 2.8*	93.9 ± 8.8*

*CSA* cross sectional area, _*a*_ thoracic aorta, _*m*_ mesenteric artery. Values are mean ± SEM (*n* = 7 for each group); *p < 0.05 vs. control group

### Effects of asiatic acid on serum Ang II, ACE and plasma NE concentrations

The levels of serum Ang II and ACE were significantly increased in MS rats compared to control rats (*p* < 0.05; Table 3). Plasma NE concentration in MS rats was also significantly higher than that of control rats. A reduction of serum Ang II, ACE and plasma NE levels in MS rats treated with asiatic acid (MS + AA group) was found (*p* < 0.05; Table 3).

**Table 3:** Effects of asiatic acid on serum Ang II, ACE and plasma NE concentrations

Parameters	Normal control	MS	MS + AA
Serum Ang II (pg/mL)	1.7 ± 0.3	3.1 ± 0.4*	2.1 ± 0.2**
Serum ACE (ng/mL)	247.5 ± 35.8	328.4 ± 14.3*	268.6 ± 26.5**
Plasma NE (ng/L)	84.9 ± 12.6	190.9 ± 39.5*	119.7 ± 21.5**

*Ang II* angiotensin II, *ACE* angiotensin converting enzyme, *NE* norepinephrine. Values are mean ± SEM (*n* = 6 for each group); *p < 0.05 vs. control group; **p < 0.05 vs. MS group

### Effects of asiatic acid on contractile responses to EFS and exogenous NE in mesenteric vascular bed isolated from MS rats

**Fig. 3:**
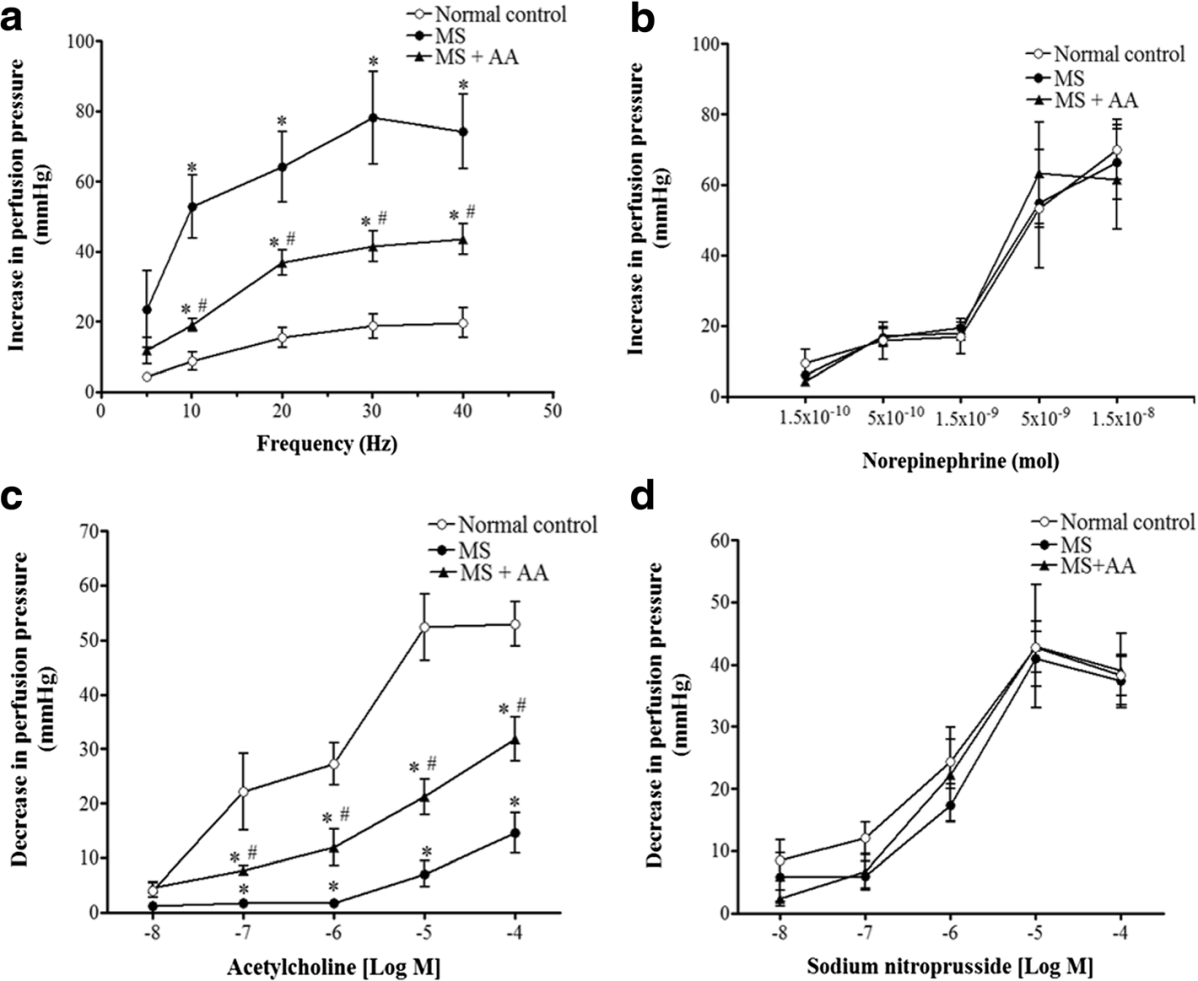
Effects of asiatic acid on vascular function in mesenteric vascular beds. Contractile responses to sympathetic nerve stimulation (**a**); exogenous norepinephrine (**b**); vasorelaxation responses to exogenous acetylcholine (**c**); sodium nitroprusside (**d**) in mesenteric vascular beds isolated from control, MS and MS + AA rats. Values are mean ± SEM (*n* = 6 for each group), *p <* 0.05. * vs. control group, ^#^vs. MS group. Normal control = normal control rats, MS = metabolic syndrome rats, MS + AA = metabolic syndrome rats treated with asiatic acid

EFS at 5–40 Hz produced an increased in perfusion pressure caused by vasoconstriction, and vasoconstriction was frequency-dependent in all preparations. A significant increase in contractile responses to EFS was observed in the mesenteric vascular bed isolated from MS rats compared to the responses in control rats (at 30 Hz, 78.2 ± 13.1 vs. 18.9 ± 3.14 mmHg, *p* < 0.01). Contractile response to EFS in MS rats-tread with asiatic acid was reduced (at 30 Hz, 41.5 ± 4.31 mmHg, *p* < 0.05) compared to the response from MS rats (Fig. 3a). However, the contractile response to exogenous NE (0.15 nmol–15 nmol) was not different across groups (Fig. 3b).

### Effects of asiatic acid on vascular responses to vasoactive agents in the rat perfused mesenteric vascular bed under raised tone conditions

Vasorelaxation responses to ACh (0.1 μM–0.1 mM) were significantly blunted in MS preparations compared to the response in preparations from control rats (ACh (10 μM), 8.2 ± 2.5 vs. 46.2 ± 6.4 mmHg) (*p* < 0.01). MS rat treated with asiatic acid (MS + AA) showed improvement of the response to ACh compared to MS rats (ACh (10 μM), 21.2 ± 3.2 mmHg) (*p* < 0.05; Fig. 3c). There was no significant difference in the vasorelaxation responses to SNP in any group, indicating normal vascular smooth muscle cell function (Fig. 3d).

### Effects of asiatic acid on vascular responses to vasoactive agents in the thoracic aorta

**Fig. 4:**
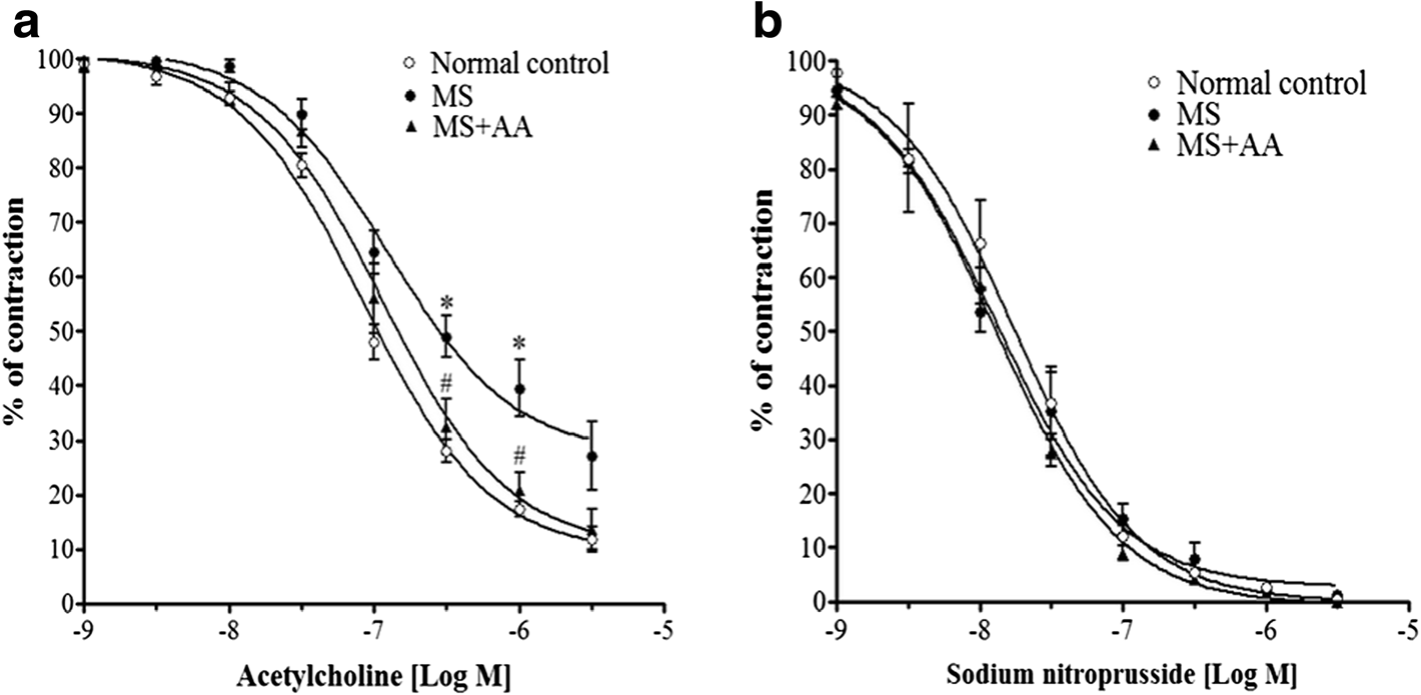
Effects of asiatic acid on vascular function in thoracic aorta. Vasorelaxation responses to exogenous acetylcholine (**a**) and sodium nitroprusside (**b**) in thoracic aorta collected from control, MS and MS + AA rats. Values are mean ± SEM (*n* = 6 for each group), *p <* 0.05. * vs. control group, ^#^vs. MS group. Normal control = normal control rats, MS = metabolic syndrome rats, MS + AA = metabolic syndrome rats treated with asiatic acid

ACh (0.01 μM–3 μM) caused endothelium-dependent relaxation in a concentration-dependent manner in all preparations (Fig. 4a). However, vasorelaxation responses to ACh were significantly reduced in MS preparations compared to the response in control group rats (ACh (1 μM), 17.4 ± 1.3 vs. 39.5 ± 5.2 % of contraction) (*p* < 0.05). An improvement of ACh-induced relaxations was seen in the aortic rings isolated from MS + AA rats (ACh (1 μM), 20.7 ± 3.3 % of contraction) (*p* < 0.05). The vasorelaxation response to SNP, an NO donor, did not differ significantly across experimental groups (Fig. 4b).

### Effects of asiatic acid on mesenteric eNOS protein expression

**Fig. 5:**
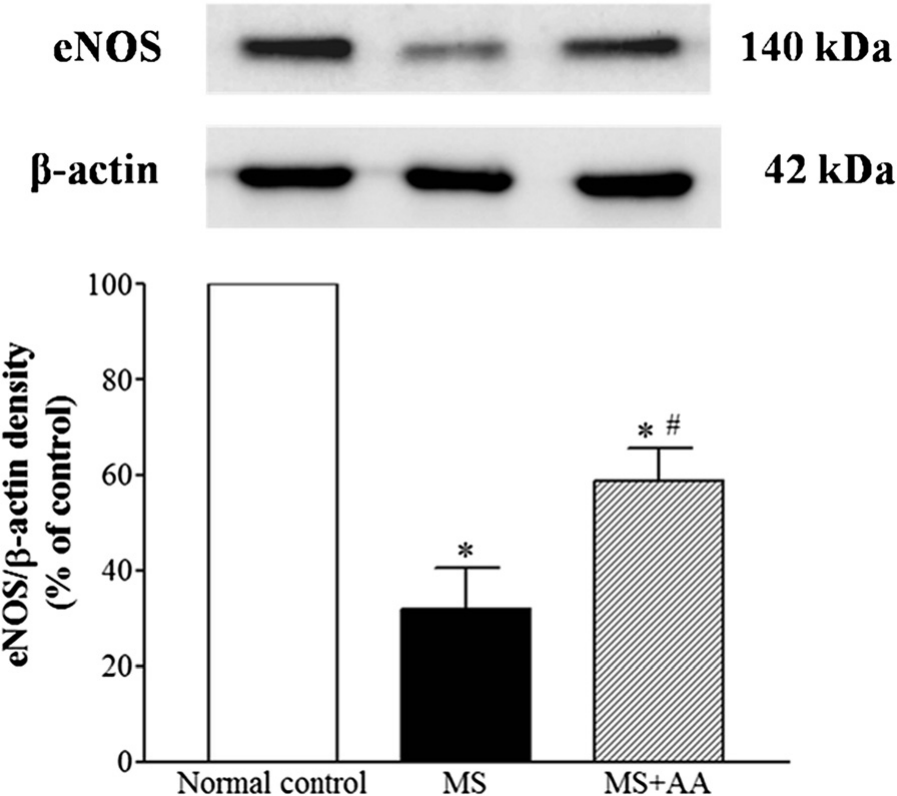
Effects of asiatic acid on eNOS protein expression in the mesenteric artery. Values are mean ± SEM (*n* = 4 for each group), *p <* 0.05. * compared with the control group, ^#^ compared with the MS group. Normal control = normal control rats, MS = metabolic syndrome rats, MS + AA = metabolic syndrome rats treated with asiatic acid

Downregulation of eNOS protein expression was found in the mesenteric artery from MS rats comparing to that of control rats (*p* < 0.001). Asiatic acid treatment (MS + AA) significantly increased the eNOS protein expression compared to that of MS rats (*p* < 0.01; Fig. 5) but these values were still lower than those in the control group.

## Discussion

The present study examined the vascular effects of asiatic acid in HCHF diet-induced MS in rats. The findings demonstrate that rats fed with a HCHF diet had signs of MS, such as high blood pressure, insulin resistance, hyperglycemia and dyslipidemia. Vascular remodeling with upregulation of AT_1_ receptor protein expression, endothelial dysfunction with downregulation of eNOS protein expression as well as increased sympathetic nerve-mediated response with increased plasma NE were also seen in MS rats. Treatment with asiatic acid for 3 weeks appeared to offset some metabolic alterations, decreased blood pressure and improved vascular function in MS rats. This response is consistent with the modulation of AT_1_ receptor/eNOS protein expression, serum Ang II and plasma NE in MS rats-treated with asiatic acid.

HCHF diet-induced MS rats presented with hypertension, dyslipidemia and insulin resistance. These signs of MS were alleviated in MS rats treated with asiatic acid for 3 weeks. There is substantial evidence to support the beneficial effect of asiatic acid in animals with these metabolic alterations. For example, we previously reported that asiatic acid with its antioxidant and anti-inflammatory activity, improved hemodynamic and metabolic alterations in diet-induced metabolic syndrome [5]. Asiatic acid has also been shown to ameliorate insulin resistance and oxidative stress and reduce inflammatory markers in mice hepatic tissues, resulting in protection from high fat diet-induced hepatic injury [32]. In streptozotocin induced diabetic rats, asiatic acid attenuated hyperlipidemia and hyperglycemiavia regulating key enzyme in lipid metabolism [33]. Other biological effects of asiatic acid have been demonstrated. Wang revealed anti-glycative effects of asiatic acid that related to decreasing oxidative stress and inflammation in human keratinocyte cells [34]. Antihypertensive effects of asiatic acid in animal model hypertension has been reported to involve the modulation eNOS/p^47phox^ protein expression [28].

We have found changes in vascular structure in both large and small arteries. Thickening of vascular wall, increased CSA and luminal area were seen in the aorta and mesenteric arteries of MS rats. Vascular remodeling results from adaptive processes as a response to hemodynamic alterations. Ang II is one important factor that promotes vascular remodeling in hypertension. Ang II acts via an intracellular signaling pathway after binding to its cellular-surface receptors on vascular smooth muscle cells [35]. We found increases in serum Ang II level as well as upregulation of AT_1_ receptor expression in the aorta of MS rats. This is consistent with several studies that report activation of the renin-angiotensin system in diet induced MS [20–22]. These reports suggest that the renin-angiotensin system contributes for the development of hypertension in this animal model. Furthermore, the alterations of renin-angiotensin system and hypertension presented in MS rats were reversed with treatment with asiatic acid. This might be caused by the anti-hyperinsulinemic and anti-hyperglycemic effects of asiatic acid, since there are substantial data to confirm that chronic hyperinsulinemia and hyperglycemia can enhance renin-angiotensin system [36]. However, treatment of asiatic acid for 3 weeks failed to improve vascular remodeling in the aorta and mesenteric arteries in MS rats. The exact mechanism whereby asiatic acid acts on vascular remodeling is unclear. Longer treatment with asiatic acid in this animal model might elucidate this.

One explanation of the high blood pressure induced by HCHF in MS rats is increased sympathetic nerve activity. This idea is supported by increases in heart rate and NE level in plasma of MS rats. Furthermore, enhancement of the contractile response to EFS in mesenteric vascular bed isolated from MS rats was observed, whereas, the contractile response to exogenous NE was not different among group of rats. This data might imply that the increased contractile response to sympathetic nerve stimulation was mediated via prejunctional site where the release of NE could be augmented. There is evidence to show the association between hyperinsulinemia and abnormal neuronal regulation of vascular tone [15, 25]. Zamami and coworkers found that the sympathetic nerve mediated-contractile response to perivascular nerve stimulation and plasma NE levels in fructose drinking rat was greater than those to in control rats. It might be associated with hyperinsulinemia induced sympathetic nerve fiber distribution [25]. Moreover, recent studies demonstrated that insulin resistance-induced hypertension could be a result of the enhancement of adrenergic nerve mediated vasoconstriction in hyperinsulinemia rats [37, 38]. In the present study, we found that asiatic acid attenuated neurogenic-mediated vasoconstriction and plasma NE which could be resulted from the alleviation of plasma insulin in the treated MS rats.

The current study showed endothelial dysfunction in the aortic ring and mesenteric vascular beds isolated from MS rats. Since the vasorelaxation response to ACh but not SNP was blunted in this animal model, these findings are consistent with previous observations that there is an impairment of endothelium dependent-vasorelaxation in thoracic aorta in MS [24, 39]. Downregulation of eNOS protein expression in the mesenteric artery of MS rats was also observed in the present study. In the vascular endothelium, it is well established that NO is synthesized from L-arginine by eNOS to control vascular tone and blood pressure [40, 41]. Thus, downregulation of eNOS protein expression may reduce the level of NO bioavailability and increase total peripheral resistance resulting in increased systolic blood pressure. Asiatic acid supplementation significantly improved vascular response to ACh in the thoracic aorta and the mesenteric vascular beds, which was associated with restoration of eNOS protein expression in the mesenteric artery. This beneficial effect of asiatic acid on eNOS protein expression in aortic tissue supports the findings of a previous study [5, 28]. These results suggest that asiatic acid ameliorates hypertension in HCHF diet-MS rats via improving the endothelial function in both conduit and resistance vessels.

## Conclusions

In summary, the present study demonstrates that asiatic acid alleviates signs of MS in HCHF diet-induced MS in rats. It reduces blood pressure by decreasing renin-angiotensin overactivity, sympathetic nerve overactivity and improving vascular function, in HCHF diet-induced MS rats.

## Ethics approval and consent to participate

All procedures on animals in this study were complied with the standards for the care and use of experimental animals and were approved by the Animal Ethics Committee of Khon Kaen University, Khon Kaen, Thailand (AEKKU 36/2555).

## Consent for publication

Not applicable.

## Additional file

Additional file 1: Energy densities in the food pellets of standard chow diet and high carbohydrate high fat (HCHF) diet. (DOCX 18 kb)

## Abbreviations

AA: asiatic acid
ACE: angiotensin converting enzyme
ACh: acetylcholine
Ang: angiotensin
AT_1_: angiotensin II receptor type 1
CSA: cross-sectional area
DP: diastolic blood pressure
EFS: electrical field stimulation
eNOS: endothelial nitric oxide synthase
FBG: fasting blood glucose
HBF: hindlimb blood flow
HCHF: high-carbohydrate high-fat
HOMA-IR: homeostasis model assessment of insulin resistance
HR: heart rate
HVR: hindlimb vascular resistance
MAP: mean arterial pressure
MS: metabolic syndrome
NE: norepinephrine
RAS: renin-angiotensin system
SNP: sodium nitroprusside
SP: systolic blood pressure
