# Supplementary material for: Asiatic acid attenuates renin-angiotensin system activation and improves vascular function in high-carbohydrate, high-fat diet fed rats
Source: BMC Complement Altern Med. 2016 Apr 27;16:123. doi: 10.1186/s12906-016-1100-6 (PMC4849098; doi:10.1186/s12906-016-1100-6)
Supplement: Additional file 1: — Energy densities in the food pellets of standard chow diet and high carbohydrate high fat (HCHF) diet. (DOCX 18 kb) [file 12906_2016_1100_MOESM1_ESM.docx]

**Additional file 1**

**Energy densities in the food pellets of standard chow diet and high carbohydrate high fat (HCHF) diet [**[**1**](#_ENREF_1)**].**

| **Macronutrient Composition** | **Standard Chow Diet** | **HCHF Diet** |
| --- | --- | --- |
| Total carbohydrates, g/100 g | 56.24 | 55.06 |
| Total fat, g/100 g | 5.78 | 18.85 |
| Protein, g/100 g | 24.76 | 8.83 |
| Crude fiber, g/100 g | 1.91 | 0.75 |
| Ash, g/100 g | 6.12 | 3.94 |
| Moisture, g/100 g | 4.40 | 13.82 |
| Energy, Kcal/100 g | 386.82 | 423.21 |

**Reference**

^1.^ Senaphan K, Kukongviriyapan U, Sangartit W, Pakdeechote P, Pannangpetch P, Prachaney P et al. Ferulic Acid Alleviates Changes in a Rat Model of Metabolic Syndrome Induced by High-Carbohydrate, High-Fat Diet. Nutrients. 2015;7(8):6446-64.
